# Nonbacterial Thrombotic Endocarditis of Bioprosthetic Aortic Valve Presenting as Cardioembolic Stroke in a Patient without Predisposing Systemic Disease

**DOI:** 10.1155/2023/5411153

**Published:** 2023-10-12

**Authors:** Samuel J. White

**Affiliations:** Robinson Research Institute, Faculty of Health and Medical Sciences, University of Adelaide, Adelaide, 5005 South Australia, Australia

## Abstract

Nonbacterial thrombotic endocarditis (NBTE) describes a cluster of noninfectious heart valve lesions and is histologically characterised by the presence of sterile platelet and fibrin-rich aggregates. Risk factors include hypercoagulable states such as active malignancy, autoimmune disorders, and prothrombotic haematological conditions. NBTE involving bioprosthetic heart valves is exceedingly rare. We present a case of a 73-year-old man with a bioprosthetic aortic valve and no NBTE risk factors who developed right-sided homonymous hemianopia during an admission for decompensated congestive cardiac failure. After detailed clinical work-up including brain MRI, the man was diagnosed with acute ischemic cardioembolic stroke involving the left posterior cerebral artery territory. He subsequently underwent successful bioprosthetic aortic valve replacement with histologic examination of the explant supporting diagnosis of NBTE. Evidence of new neurological deficit or disturbance in patients with prosthetic heart valves should prompt investigation for cardioembolic stroke. Whilst rare, NBTE should be considered as a differential diagnosis for cardioembolic stroke, even in patients without predisposing systemic disease.

## 1. Introduction

Nonbacterial thrombotic endocarditis (NBTE) describes a cluster of noninfectious heart valve lesions and is histologically characterised by the presence of platelet and fibrin-rich aggregates devoid of bacteria and inflammatory cells [1]. Risk factors for NBTE include hypercoagulable states such as active malignancy [2], autoimmune disorders, and prothrombotic haematological conditions [3]. NBTE is often asymptomatic until cardioembolic phenomena arise with the brain, kidney, spleen, and mesentery being commonly affected sites [1]. Presentation with heart failure symptoms due to valvular insufficiency or stenosis due to large vegetation size is uncommon [4]. Diagnosis in asymptomatic individuals is typically made at autopsy, with an estimated prevalence of 0.3-9.3% in autopsied individuals [5]. NBTE involving bioprosthetic valves is exceedingly rare. Only two cases involving patients without predisposing underlying systemic disease have previously been reported [6, 7].

## 2. Case Presentation

The patient is a 73-year-old man with a background of ischemic heart disease, aortic regurgitation requiring a 27 Mosaic valve bioprosthetic aortic valve implantation in 2009, type 2 diabetes mellitus, hypertension, hypercholesterolemia, stage 4 chronic kidney disease, and peptic ulcer disease. His significant medication history included lifelong clopidogrel therapy for ischemic heart disease; amlodipine, ramipril, and prazosin for hypertension; atorvastatin and ezetimibe for hypercholesterolemia; and metformin, gliclazide, dulaglutide, and insulin aspart with degludec for type 2 diabetes mellitus. The patient had a family history of cardiovascular disease with both parents dying from myocardial infarction in their 60s.

The patient initially presented with a 2-week history of worsening exertional dyspnoea, orthopnoea, and intermittent presyncope in the absence of any obvious precipitating causes. ECG demonstrated sinus rhythm with borderline left bundle branch block, which was preexisting. There were no dynamic ischemic changes. He was admitted for intravenous diuresis and further work-up. A few days into his admission, the patient awoke with new right-sided visual disturbance. His vital signs were within normal limits with blood pressure of 138/86 mmHg and heart rate of 86 beats per minute. The patient was oriented to time, place, and person. Neurological examination demonstrated normal tone and reflexes, intact cranial nerves, and no abnormality of power or sensation in the limbs. However, visual field assessment demonstrated a right-sided homonymous hemianopia. Cardiovascular examination was unremarkable with a regular rate and no rub, gallop, or murmur. There was evidence of mild fluid overload with scattered coarse crepitations at the lung bases and pitting oedema to the ankles bilaterally. The abdomen was soft, nontender, and not distended. There were no Janeway lesions, Osler's nodes, petechiae, or splinter haemorrhages of the fingernails. ECG demonstrated sinus rhythm and no dynamic ischemic changes.

The patient's haemoglobin was 102 g/L, and white cell count elevated at 15.3 × 10^9^/L. C-reactive protein was also increased at 121 mg/L. Five sets of blood cultures returned negative results and serology for *Coxiella* and *Bartonella* were unremarkable. Coagulation studies including prothrombin time (15), international normalised ratio (0.9), and activated partial thromboplastin time (31) were unremarkable, and blood film analysis was normal. An additional prothrombotic panel comprising protein C, protein S, and factor V Leiden was also normal.

**Figure 1:**
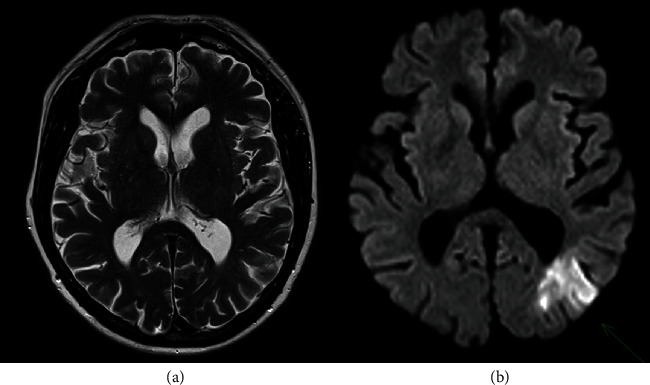
Axial diffusion-weighted brain MRI (b) demonstrating diffusion restriction involving the left occipital lobe at the junction of the left posterior cerebral artery and left middle cerebral artery territories (indicated by green arrow). Axial T2-weighted brain MRI (a) was unremarkable and did not demonstrate evidence of haemorrhagic transformation or previous stroke.

**Figure 2:**
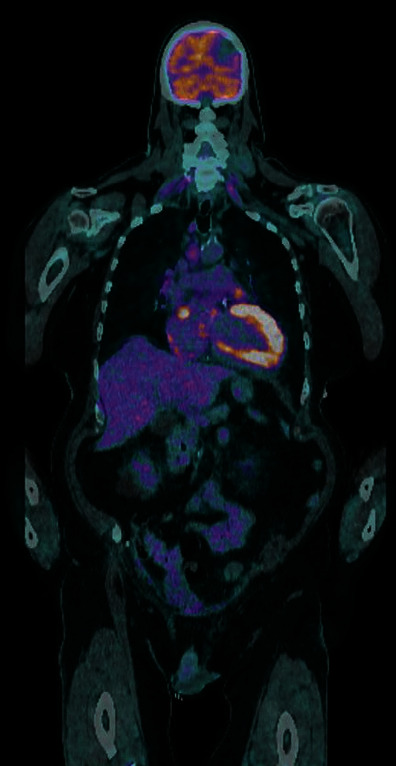
Coronal cardiac positron emission tomography (PET) image demonstrating absence of FDG avidity at the bioprosthetic aortic valve. Physiological FDG avidity is noted throughout the myocardium of the left ventricular wall.

Diffusion-weighted brain MRI demonstrated diffusion restriction involving the left occipital lobe at the junction of the left posterior cerebral artery and left middle cerebral artery territories (Figure 1). There was no evidence of haemorrhagic transformation and no old T2 infarcts suggestive of previous cardioembolic stroke. The patient proceeded to have a transoesophageal echocardiogram (TOE), which showed a 10 × 7.5 mm heterogenous mobile echogenicity attached to the bioprosthetic aortic valve with concomitant degeneration of the leaflets. There was moderate aortic stenosis and severe paravalvular aortic regurgitation and no evidence of valvular abscess. Left ventricular stroke volume was 77 mL with an ejection fraction of 45%, and left ventricular outflow tract velocity was 0.82 metres per second. Cardiac positron emission tomography (PET) imaging did not demonstrate FDG avidity at the bioprosthetic aortic valve (Figure 2).

A provisional diagnosis of NBTE was based on presentation of acute ischemic stroke with a non-FDG-avid heterogenous bioprosthetic aortic valve vegetation and negative blood cultures. Embolization of a calcific fragment of the degenerated bioprosthetic valve remained a potential differential diagnosis. Blood culture-negative infective endocarditis was considered less likely as the patient remained afebrile and did not have recent antibiotic exposure. Autoimmune aetiology was excluded on the basis of lack of personal or family history of autoimmune disease, as well as an unremarkable blood panel including antinuclear antibodies and antiphospholipid antibodies (specifically, anti-b2 glycoprotein, lupus anticoagulant and anticardiolipin). A malignant aetiology was excluded on the basis of a negative CT chest, abdomen, and pelvis, as well as an unremarkable panel of tumour markers including alpha-fetoprotein (AFP), beta-human chorionic gonadotropin (beta-hCG), cancer antigen 19-9 (CA 19-9), chromogranin A, and carcinoembryonic antigen (CEA). A prothrombotic disorder was excluded on the basis of the patient's normal coagulation profile, absence of personal or family history of prothrombotic disorder, and lack of peripheral stigmata of prothrombotic disorder.

In light of the vegetation size, active embolization, and evidence of severe valvular degeneration on TOE, the decision was made to proceed to emergent redo bioprosthetic aortic valve replacement (25 Perimount Magna Ease). Intraoperatively, severe degeneration of the patient's original bioprosthetic aortic valve was noted with significant paravalvular regurgitation near the left coronary cusp. There was no evidence of active infection. Histologic examination of the explant was consistent with NBTE, demonstrating platelet and fibrin-rich thrombus attached to the valve leaflets. There was no evidence of active inflammation, dystrophic calcification, or pannus formation. The absence of inflammation or eosinophils (and serum eosinophils within normal range) helped to exclude porcine valve allergic endocarditis, which is a very rare cause of endocarditis that has been described in a handful of patients with porcine bioprosthetic heart valves [8]. Tissue culture did not grow any microorganisms. Specifically, periodic acid-Schiff (PAS) with diastase stain was negative, which helped to exclude Whipple's endocarditis.

Postoperatively, the patient was prescribed warfarin for a 3-month course (target INR 2.0-3.0) with bridging heparin to cover for risk of thromboembolism. Following this, the patient was switched back to lifelong clopidogrel therapy.

**Figure 3:**
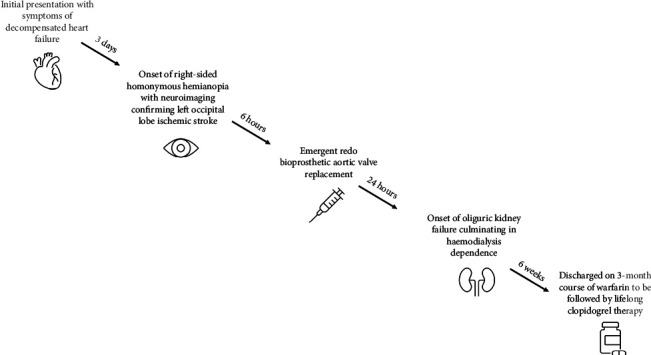
Flowchart demonstrating timeline of patient's clinical course.

The patient's postoperative course was complicated by oliguric acute kidney injury culminating in haemodialysis dependence. Imaging did not demonstrate evidence of renal infarct. The patient was discharged six weeks after his redo bioprosthetic aortic valve replacement (Figure 3).

## 3. Discussion

Whilst not fully elucidated, the pathogenesis of NBTE is thought to involve endothelial damage due to factors such as hypoxia, active malignancy, prothrombotic haematological disease, and autoimmune disease [1]. Endothelial damage exposes subendothelial connective tissue to circulating platelets, promoting formation of platelet and fibrin-rich thrombus [1]. Given the absence of other risk factors (malignancy, prothrombotic disorder, and autoimmune disease), we hypothesised that hypoxia was the most likely cause of NBTE in our patient who was admitted with congestive cardiac failure symptoms. However, it is also likely that the patient's endocarditis contributed to their heart failure.

The clinical presentation of NBTE is often similar to that of infective endocarditis. Negative blood cultures and the absence of fever and peripheral stigmata of infective endocarditis are more consistent with NBTE or embolism of a fragment from a degenerated valve compared to infective endocarditis. Ultimately, histologic examination is required to definitively diagnose NBTE.

Historically, recurrent embolization has been viewed as a hallmark feature of NBTE with an estimated prevalence of 50% [5]. In a study assessing diffusion-weighted brain MRI patterns in patients with NBTE presenting with acute stroke, 100% (9/9) of patients had multiple cerebral emboli. Interestingly, our patient only had a single site of cerebral embolization. NBTE involving a bioprosthetic aortic valve in the absence of predisposing systemic disease has previously been recorded in two patients. One patient had bilateral renal infarcts and died following pulseless electrical activity arrest [6]. The other did not have any evidence of embolic phenomena and recovered following redo bioprosthetic aortic valve replacement [7].

Management of predisposing systemic disease and systemic anticoagulation has traditionally formed the basis of treatment for NBTE. Indications for operative management include acute heart failure, valvular rupture, and prevention of recurrent embolization, although there are no formal guidelines [1, 9, 10]. Additional complexity arises in cases where, similar to our patient, patients do not have modifiable risk factors for NBTE. Ram et al. raise the question of whether there would exist any benefit in choosing a mechanical valve instead of a redo bioprosthetic valve in this cohort of patients [7]. Unfortunately, there is insufficient data to address this question at present due to the rarity of NBTE in patients with bioprosthetic heart valves. We agree with Ram et al.'s assertion that patients with bioprosthetic heart valves and risk factors for NBTE be anticoagulated [7].

Although rare, NBTE should always be considered in patients with bioprosthetic heart valves presenting with symptoms potentially attributable to decompensation or cardioembolic phenomena, even in the absence of predisposing systemic disease.

## Data Availability

The deidentified data presented in this case report were obtained from an electronic medical records system and are therefore not able to be made publicly available.
